# Heteronuclear Dual Metal Atom Electrocatalysts for Water-Splitting Reactions

**DOI:** 10.3390/molecules29081812

**Published:** 2024-04-16

**Authors:** Lu Lu, Xingcai Wu

**Affiliations:** 1 Paris Curie Engineer School, Beijing University of Chemical Technology, Beijing 100029, China; 2 Key Laboratory of Mesoscopic Chemistry of MOE, School of Chemistry and Chemical Engineering, Nanjing University, Nanjing 210093, China

**Keywords:** heteronuclear dual metal atoms, electrocatalysts, water-splitting, electrocatalytic mechanism

## Abstract

Hydrogen is considered a promising substitute for traditional fossil fuels because of its widespread sources, high calorific value of combustion, and zero carbon emissions. Electrocatalytic water-splitting to produce hydrogen is also deemed to be an ideal approach; however, it is a challenge to make highly efficient and low-cost electrocatalysts. Single-atom catalysts (SACs) are considered the most promising candidate to replace traditional noble metal catalysts. Compared with SACs, dual-atom catalysts (DACs) are capable of greater attraction, including higher metal loading, more versatile active sites, and excellent catalytic activity. In this review, several general synthetic strategies and structural characterization methods of DACs are introduced, and recent experimental advances in water-splitting reactions are discussed. The authors hope that this review provides insights and inspiration to researchers regarding DACs in electrocatalytic water-splitting.

## 1. Introduction

Nowadays, fossil fuels are the main energy source for humanity. The combustion of fossil fuels produces carbon dioxide and leads to the greenhouse effect. With the shortage of traditional fossil fuels and the aggravation of environmental problems, it is pivotal to develop clean, cheap, convenient, and sustainable alternative energy.

Hydrogen energy is considered an ideal substitute for conventional fuels due to its widespread sources, high calorific value of combustion, and zero carbon emissions [1]. Currently, approximately 95% of the world’s hydrogen is generated from natural gas and fossil fuels through an energy-intensive process [2]. Compared with traditional technologies, electrocatalytic water-splitting systems can realize the use of renewable energy (e.g., solar energy) for uninterrupted and efficient hydrogen production. Thus, they have gained ever-rising attention in recent years [3].

The electrocatalytic water-splitting comprises a cathodic hydrogen evolution reaction (HER) and an anodic oxygen evolution reaction (OER) [4,5]. The efficiency and activity of the water-splitting system depend on the electrocatalysts. To date, only less than 4% of the hydrogen is produced by the electrocatalytic water-splitting systems [6] due to the high cost and unsatisfied economic benefits compared with conventional fossil energy production. Currently, metal-based electrocatalysts are widely applied in both HER and OER. For example, Pt group electrocatalysts are considered the benchmark for HER because of their excellent activities [7,8]. Noble metal-based electrocatalysts were reported to exhibit superior OER performances [9,10]. However, the high price and natural scarcity of noble metals hinder their practical applications for water-splitting electrocatalysts. Reducing the content of the noble metal in metal-based electrocatalysts is an effective and straightforward way to reduce cost. When the noble metal content is reduced, the utilization of the noble metal component should be increased to maintain the intrinsic activity. Therefore, many studies focus on increasing metal centers with low coordination by reducing the size of nanoparticle catalysts. In these studies, atomic-level catalysts have become a hotspot.

Single-atom catalysts (SACs) have atomic active sites dispersed on the surface of supports or alloying with another material [11]. By merit of maximum atom utilization and adjustable coordination, it has been widely investigated in the field of electrocatalysis since it was first reported by Zhang et al. in 2011 [12,13,14,15,16,17,18,19]. At the same time, SACs often suffer from the predicament of low loading (<1.5 wt%), which may limit the overall activity. Furthermore, some multi-electron-proton coupling reactions are limited at single-atom sites, and the random dispersion of SACs leads to an unsatisfied synergy [20].

As an extension of SAC, heteronuclear dual-atom catalysts (DACs) with co-doped multi-metals have drawn great attention in recent years. They retain the advantages of SACs and overcome the limitations of SACs, which can increase the metal loading, break the linear scaling relations [21], and adjust the electronic structure of the catalysts [22]. Meanwhile, the cooperation between the two metal components of DACs can generate a synergistic effect to enhance the intermediates’ adsorption and modulate reaction pathways due to the chemical/structural flexibility of active sites [23].

Although numerous high-quality reviews focused on DACs, including the definition of the DACs, the micro-structures, and different applications of DACs in various fields, establishing them as the next frontier after SACs [24,25,26,27,28], the discussion on the design of heterogeneous DACs for water-splitting is not sufficient. Therefore, it is valuable to review the progress of DACs in water-splitting reactions.

In this review, we present a comprehensive overview of the rational design and development of heterogeneous DACs in water-splitting reactions. First, we briefly introduce some synthetic strategies for heterogeneous DACs. Subsequently, the typical characterizations of DACs are summarized. Then, the recent experimental advances in water-splitting reactions are discussed in detail. Finally, this review provides a summary of the problems and challenges regarding the water-splitting technologies.

## 2. Synthetic Strategies for DACs

Efficient strategies were adopted to synthesize well-defined DACs. Each technique brings its advantages and drawbacks. We will introduce several common approaches to construction DACs for water-splitting reactions.

### 2.1. High-Temperature Pyrolysis

High-temperature pyrolysis is a general strategy to synthesize atomic-level reactive sites. The pyrolysis temperature and atmosphere composition are crucial for the formation of DACs. Benefiting from the tunable pore structure and large surface area, metal–organic frameworks (MOFs) and zeolitic imidazolate frameworks (ZIFs) have been widely used as precursors to fabricate DACs for water-splitting reactions.

**Figure 1:**
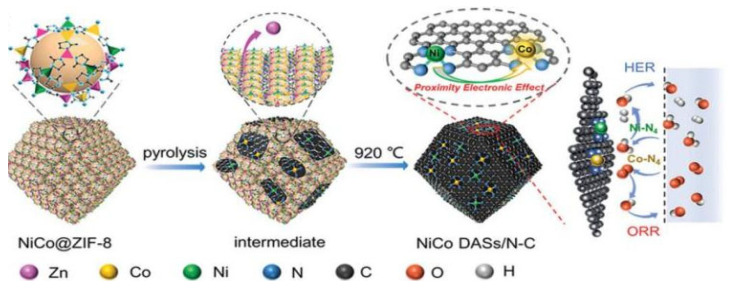
Schematic illustration of the synthesis process of NiCo DASs/N-C. Reproduced with permission [29]. Copyright 2022, Wiley-VCH GmbH.

For example, Pan et al. reported a MOF-assisted host–guest strategy to prepare Ni-Co DACs, as shown in Figure 1 [29]; the Ni and Co atoms were sealed in the cage of ZIF-8, which served as a host with suitable pore size. Then, the NiCo@ZIF-8 precursor was pyrolyzed under N_2_ at 920 °C to generate Ni-N_4_ and Co-N_4_ sites embedded into a 3D carbon substrate. During the process, the ZIF-8 was carbonized to form the N-C substrate to provide anchor sites; the anchored Co-N_4_ can adsorb and activate O_2_, which promotes the formation of key intermediate and accelerates the reaction kinetics. Meanwhile, the adjacent Ni-N_4_ can further improve the activity of Co-N_4_ and endow the electrocatalyst with outstanding HER activity in both acid and alkaline solutions. The NiCo DACs require overpotentials of 189 and 260 mV to reach the current density of 10 mA cm^−2^ in 1 M KOH and 0.5 M H_2_SO_4_, respectively.

In the study of Jia et al. [30], porous graphitic g-C_3_N_4_ was synthesized as a precursor, and then a Ni precursor, polydopamine, and phthalocyanine iron were coated on the surface of porous graphitic g-C_3_N_4_ in that order. After pyrolyzing under 900 °C, the porous graphitic g-C_3_N_4_ formed porous carbon nanosheets, the Fe/N_4_ and Ni/N_4_ sites formed on both sides of the carbon nanosheets with Janus structure (FeNi_jns_/NC). During this process, the g-C_3_N_4_ was used as a sacrificial template to form the lamellar and porous morphology, which can bond and stabilize the Ni/Fe SAs and avoid the migration of the active sites. Experiments indicate that the overpotential of FeNi_jns_/NC is 440 mV at 10 mA cm^−2^, and the Tafel slope is fitted to be 106 mV dec^−1^, demonstrating that FeNi_jns_/NC possesses superior OER activity.

In short, high-temperature pyrolysis can regulate the dispersion of metal sites easily, and the structure and the active sites of DACs can be well-defined. However, some active metals may be incorporated into the bulk of the substrate materials, which can hardly be accessible during electrocatalysis. Meanwhile, undesirable impurities may be generated during the pyrolysis, which usually requires an additional etching process.

### 2.2. Wet Chemistry Impregnation

The wet chemistry impregnation method is low-cost and straightforward. The DACs were prepared by introducing the precursors to the surface of the matrix and then post-treatment, such as reduction or thermal treatment.

**Figure 2:**
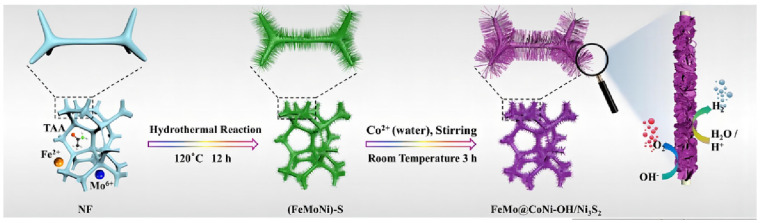
Schematic depiction of the synthesis route for FeMo@CoNi-OH/Ni_3_S_2_. Reproduced with permission [31]. Copyright 2023 Elsevier B.V.

Li et al. prepared FeSO_4_·7H_2_O and Na_2_MoO_4_·2H_2_O solutions as metal precursors, then the as-obtained solution and a piece of Ni foam were transferred to an autoclave with a Teflon liner [31]. The reaction was kept at 120 °C for 24 h to synthesize FeMo dual-site catalysts (FeMo@CoNi-OH/Ni_3_S_2_), as shown in Figure 2. The electrocatalytic performance of FeMo@CoNi-OH/Ni_3_S_2_ was then investigated in both acidic and alkaline solutions. It exhibited excellent HER performance with 89 and 177 mV to reach 10 mA cm^−2^ in 1 M KOH and 0.5 M H_2_SO_4_, respectively, indicating it is more favorable for HER compared with the other contrast samples. The successful synthesis of Fe and Mo single atoms can provide atomic sites, and the difference in electronegativity between Fe and Mo led to charge rearrangement and a charge compensation effect, thus improving the electrochemical performance.

The wet chemistry impregnation usually adopts a certain amount of precursor to generate atomically dispersed metal sites. The type and concentration of the precursor can be modulated to prepare desired DACs; however, it is difficult to precisely regulate the location of the active sites, as some metal atoms may aggregate to form bulk sites.

### 2.3. Atomic Layer Deposition (ALD)

The ALD approach generally utilizes a gas-phase reaction of metal precursors with a solid substrate to synthesize DACs. It is a gas-phase deposition strategy to deposit the thin films onto the substrate accurately.

For example, Hu et al. used (methyl cyclopentadienyl)-platinum (MeCpPtMe_3_) and O_3_ as precursors and used high-purity N_2_ as purge gas and carrier gas; an N-doped carbon substrate was loaded on aluminum foil and then placed into the ALD chamber. After 50 cycles of ALD, PtNi-NC was obtained [32]. The electrochemical measurements indicated that PtNi-NC has an overpotential of 30 mV to achieve the current density of 10 mA cm^−2^ and a Tafel slope of 27 mV dec^−1^ in acidic solution, which is lower than those of 20 wt% Pt/C and other contrast electrocatalysts. The superior electrocatalytic performance of PtNi-NC can be attributed to the dual atoms, which have a synergistic effect by optimizing the local electronic structure and the charge distribution. The ALD technique is beneficial for controlling the composition and structure of the DACs, but it requires special equipment and harsh reaction conditions.

### 2.4. Template Assisted

The template-assisted method usually utilizes precursors to act as a template or pattern to synthesize DACs. For example, a SiO_2_ template can be etched by NaOH solution, which is applied to construct a hollow catalyst. Ma et al. synthesized Ni-N_4_ and Fe-N_4_ dual single-atomic sites with a template-assisted strategy [33]. Firstly, the positively charged SiO_2_ nanospheres were adopted as a hard template, and then electronegative [Ni(CN_4_)]^2−^ was chosen to form Ni-N_4_ single-atom sites. Next, the formed SiO_2_@[Ni(CN_4_)]^2−^ intermediate was encapsulated with graphene oxide (GO) to form SiO_2_@[Ni(CN_4_)]^2−^@GO. The iron (II) phthalocyanine (FePc) was then adsorbed on the outside of SiO_2_@[Ni(CN_4_)]^2−^@GO to obtain SiO_2_@[Ni(CN_4_)]^2−^@GO@FePc. Thereafter, the SiO_2_@[Ni(CN_4_)]^2−^@GO@FePc was treated at 700 °C and etched with NaOH solution to remove the SiO_2_ template, eventually leading to the formation of a Ni-N_4_ and Fe-N_4_ dual single-atomic site. This work successfully synthesizes the Janus hollow spheres derived from different single-atom catalysts, respectively, on the inner and outer surfaces. The OER polarization curves in O_2_-saturated 0.1 M KOH indicate that the Ni-N_4_/GHSs/Fe-N_4_ has a low overpotential of 0.39 V to reach a current density of 10 mA cm^−2^, which mainly originates from the Ni-N_4_/GHSs sites.

In other works, porous carbon [34], the metal–organic framework (MOF) [35], and a salt template [36] were also investigated to fabricate the template. It indicates that the template-assisted method can synthesize a porous micro-structure but, sometimes, the removal of some templates may cause damage to the nanostructure [37].

### 2.5. Ball Milling

The ball milling techniques can mix different precursors at atomic scales homogeneously. For example, Liu et al. employed the ball milling method to synthesize CuCo/NSC electrocatalysts [38]. First, metal precursors, nitrogen precursors, and sulfur precursors were ball-milled to generate the CuCo-NS bulk complex. The content of nitrogen and sulfur can be modulated by adjusting the mass ratio of the precursor. Then, the ball milling process was conducted to generate CuCo-NS, which dispersed onto the carbon black support uniformly. After pyrolyzing under an argon atmosphere at a high temperature, a pack of CuCo DACs was obtained. The detailed experiments and theoretical validation imply a coordinated atom (heteroatom N and S) surrounds CuCo DACs in various orbitals coordination shells. Subsequently, The HER performance of CuCo-DACs was investigated in 1 M KOH. One of the as-prepared DACs, which was denoted as CuCo/NSC1, displayed an overpotential of 159 mV at 10 mA cm^−2^ and a small Tafel slope of 75.9 mV dec^−1^. The distinct coordination environment of CuCo DACs can be attributed to their different electrochemical activities. The introduction of S into coordination shells can lead the metal atoms to attain higher valence states, proving advantageous for their reaction with ΔGH* in HER.

Except for the above-mentioned methods, ion exchange [39], atom trapping [40], the electrochemical method [41], and chemical vapor deposition [42] are also employed to prepare various DACs. But there remain some challenges, such as how to synthesize DACs with both high productivity and precise location. Only by solving these problems can the preparation of DACs become easier and accessible.

## 3. Characterizations of DACs

Characterization techniques are imperative for validating the structure–activity relationship of DACs. Tradition characterization techniques, including scanning electron microscopy (SEM), X-ray diffraction (XRD), and low-magnification transmission electron microscopy (TEM), can provide the basic crystal structure of nanomaterials. Thus, advanced characterization is crucial to identify the geometric and electronic configuration of the DACs. Various techniques were employed to confirm the existence of DACs. Here, we mainly focus on two typical techniques, high-resolution aberration-corrected high-angle annular dark-field scanning transmission electron microscopy (HAADF-STEM) and X-ray absorption near-edge structure (XANES) spectroscopy, to recognize the nature of DACs.

### 3.1. High-Angle Annular Dark-Field Scanning Transmission Electron Microscopy (HAADF-STEM)

The morphology and structure of DACs can be directly observed through electron microscopy. In particular, the aberration-corrected (AC) high-angle annular dark-field scanning transmission electron microscopy (HAADF-STEM) mode is widely applied for the characterization of DACs. It can scatter at high angles and is particularly sensitive to heavy atoms; the DACs can be clearly observed from the bright spots on the support. Usually, the heavier metal atoms with a higher Z number display bright dots in dark-field images [43].

**Figure 3:**
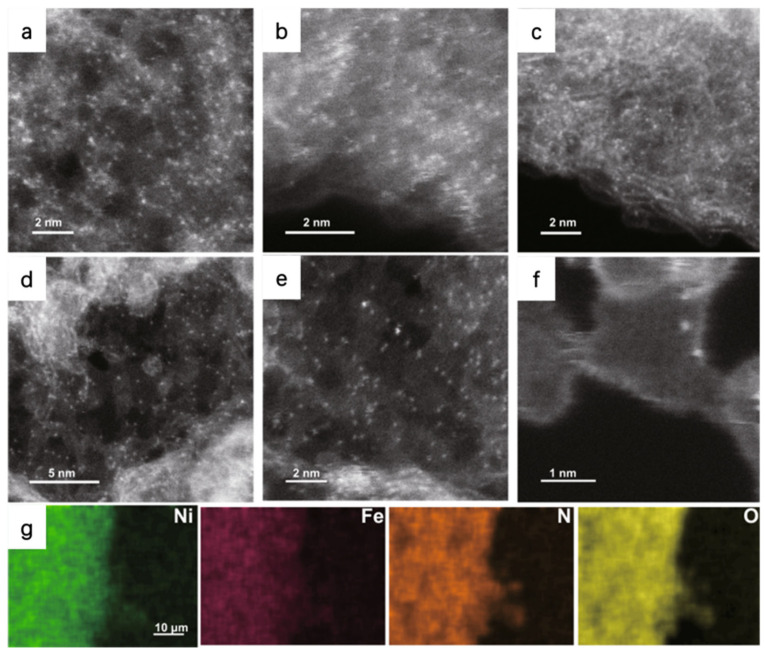
HAADF-STEM images of (**a**) Ni-CNG, (**b**) Co-CNG, and (**c**) Fe-CNG. (**d**–**f**) HAADF-STEM images of NiFe-CNG at different magnifications. The uniformly distributed bright dots represent metal atoms. (**g**) EDS mapping of NiFe-CNG recorded along with the SEM images. Reproduced with permission [44]. Copyright 2021 Springer Nature.

For example, Patzke et al. used the HAADF-STEM to observe the atomic dispersion of Ni and Fe atoms in the N-doped graphene nanosheets (NiFe-CNG); the bright dots imply the metal atoms are homogeneously dispersed on the substrate (Figure 3). Energy dispersive X-ray spectroscopy (EDS) mapping in HAADF-STEM is applied to further identify the atomic-resolution chemical composition and distribution. EDS confirms the uniform dispersion of metal, N, and O atoms in their as-prepared samples [44]. By modulating the molar ratio of Ni/Fe, the NiFe-CNG shows the best activity with an overpotential of 270 mV at the current density of 10 mA cm^−2^ and the lowest Tafel slope of 60 mV dec^−1^ in 0.1 M KOH. Combining experimental results and DFT calculations, it can be concluded that the OER benefits from the electronic interplay of the Ni-O-Fe atomic sites. The Ni/Fe dual atom can optimize local electronic structures and enhance the adsorption energies of intermediates, leading to a dual-site reaction pathway with the participation of both Ni and Fe.

The non-noble metal can also be recognized by the HAADF-STEM. In the study by Liu et al., the HAADF-STEM confirms the presence of both single atoms and dual single atoms in Fe_3_Co_7_-NC. The corresponding elemental mapping images show that Fe, Co, N, and C are uniformly dispersed over the entire matrix [45]. The Fe_3_Co_7_-NC exhibits superior OER performance with an overpotential of 343 mV and a corresponding Tafel slope of 68 mV dec^−1^ in 0.1 M KOH, indicating the synergistic effect of Fe-Co dual atoms. Combined with first-principles calculations, it suggests that the interaction between Fe and Co to form the Fe-O-O-Co bond enhances the formation of key intermediates for the OER.

Nowadays, the HAADF-STEM has become a unique and effective tool for the identify of DACs due to its structural visualization, but it is difficult to distinguish the atomic sites in different depths. It usually needs to be combined with other characterization techniques to recognize the DACs.

### 3.2. X-ray Absorption Spectroscopy

The X-ray absorption spectrum (XAS) is another useful tool to characterize the DACs. It provides a variety of information, including valences, coordination configurations, and structures. The X-ray absorption near edge structure (XANES) indicates the oxidation state of the materials, and the extended X-ray absorption fine structure (EXAFS) shows the coordination environment of the catalyst through Fourier or wavelet (FT or WT) analysis and EXAFS fitting [25].

**Figure 4:**
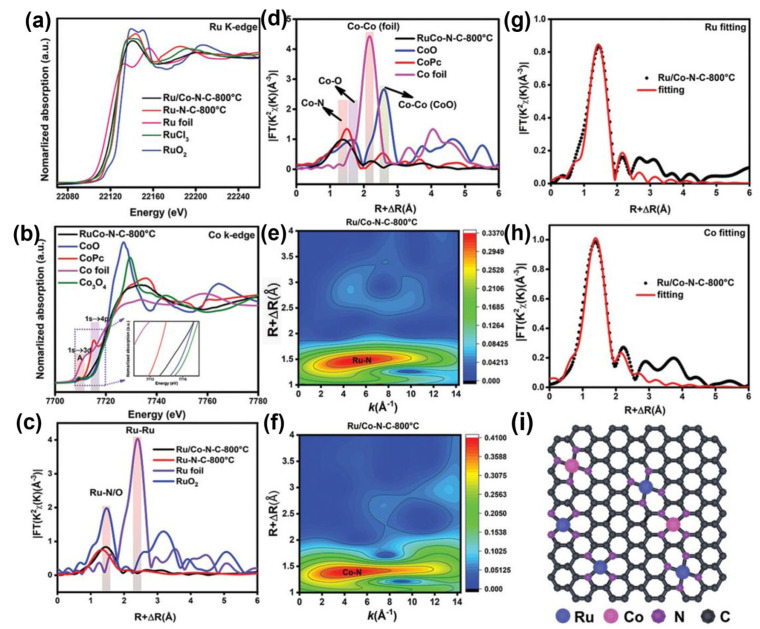
(**a**) Ru K-edge XANES spectra, (**b**) Co K-edge XANES spectra, (**c**,**d**) the corresponding Fourier transforms of EXAFS spectra of the samples, (**e**,**f**) wavelet transform of Ru and Co in Ru/Co-N-C-800 °C, respectively, (**g**,**h**) Ru K-edge and Co K-edge FT-EXAFS and the corresponding EXAFS fitting curves at R space for Ru/Co-N-C-800 °C, (**i**) structural model of Ru/Co-N-C-800 °C. Reproduced with permission [46]. Copyright 2022 Wiley-VCH GmbH.

Zhao et al. carried out XAS to observe the chemical states and coordination configurations of Ru/Co dual-sites anchored on N-doped carbon (Ru/Co-N-C-800 °C) [46]. In their XANES spectra, as shown in Figure 4, the near-edge absorption energy of the Ru K-edge was located between the Ru foil and RuO_2_, which is close to the RuCl_3_, suggesting the main valence state of Ru in Ru/Co-N-C-800 °C is about +3. Compared with RuO_2_, a pre-edge peak of Ru in Ru/Co-N-C-800 °C is related to the transition of Ru 1 s to the unoccupied Ru 4d level [47]. Additionally, the energy of Ru K-edge spectra of Ru/Co-N-C-800 °C decreased when compared with the Ru-N-C-800 °C, implying the valence of Ru decreases after introducing Co sites. As shown in Figure 4b, the normalized Co adsorption spectrum for Co K-edge spectra in Ru/Co-N-C-800 °C is positioned between CoO and Co foil, implying the oxidation of Co in Ru/Co-N-C-800 °C. A pre-edge peak (A) at ~7709.6 eV can be assigned to the dipole-forbidden but quadrupole-allowed transition, referring to the 3d and 4p orbital hybridization of the Co atoms [48]. The Fourier-transformed (FT) k^3^-weighted EXAFS (FT-EXAFS) was further conducted. The dominant peak for Ru/Co-N-C-800 °C can be assigned to the Ru-N/C bond; there are no Ru-Ru peaks that can be detected in the Ru/Co-N-C-800 °C. In the FT-EXAFS of Co K-edge, the peaks at ~2.2 Å exclude the presence of metal–metal bonds, confirming the isolation of Ru and Co atoms. The WT of K^3^-weighted EXAFS spectra further confirm the isolation of Ru and Co atoms. The only dominant peak for Ru at 4.3 Å in Ru/Co-N-C-800 °C is ascribed to the Ru-N structure. Similarly, only Co-N paths are detected, confirming the presence of Co-N bonds.

The above analysis of composition and atomic structure confirms the presence of both Ru and Co single atoms. The HER activity of Ru/Co-N-C-800 °C was then investigated. The overpotential of Ru/Co-N-C-800 °C to deliver a current density of 10 mA cm^−2^ is 19 and 17 mV in 1 M KOH and 0.5 M H_2_SO_4_, even outperforming commercial Pt/C under the same conditions. DFT calculations were conducted to validate the synergistic effect between Ru-N_4_ and Co-N_4_ sites. It indicates that the interaction between Ru and H intermediate can be weakened by CO-N_4_ sites and achieve remarkable HER activity.

In another work by Lee et al., the XANES and EXAFS were conducted to investigate the coordination environment of the Fe and Co species of the FeCo-N_4_-hollow carbonized shell (FeCo-N_4_/HCS) [49]. Based on the Fe K-edge XANES, FeCo-N_4_/HCS shows the position of the absorption threshold between Fe_2_O_3_ and iron phthalocyanine (FePc), indicating the average valence state of Fe situates between +2 and +3 [50], which can be caused by the drawing of Fe electrons to the adjacent N atoms [51]. In the FT-EXAFS, the main peak in FeCo-N_4_/HCS is similar to the FePc main peak, suggesting the dominant Fe-N coordination [52]. The EXAFS fitting exhibits that the average coordination number of Fe-N was 4.7, implying that each Fe atom is coordinated to five or four N atoms. WT-EXAFS further demonstrates the single-atom dispersion of Fe. On the other hand, the K-edge XANES of Co displays that the valence state of Co in FeCo-N_4_/HCS is ~+2 [53]. In the Co K-edge FT-EXAFS profile, the main peak can be attributed to the coordination of Co-N, suggesting the single-atomic state of Co species. The EXAFS fitting curves further indicate the single-atom Co-N_4_ model. The OER activity of FeCo-N_4_/HCS was tested in 1 M KOH. The overpotential is 391 mV, which delivers a current density of 10 mA cm^−2^, and the Tafel slope is 78.52 mV dec^−1^, indicating the superior OER kinetics and interfacial response. The outstanding electrochemical performance of FeCo-N_4_/HCS can be attributed to the highly dispersed atomic sites and hollow structure of carbonized shells, which can increase the electrocatalytic active surface area and accelerate the mass and charge transfer.

Notably, the information deduced from XAS is an average result, indicating that an inaccurate structure may be obtained if the materials have various coordination configurations. Thus, the XAS should unite with other tools to confirm the real structural information.

Additionally, X-ray photoelectron spectroscopy (XPS), Mössbauer spectroscopy, and Fourier-transform infrared (FTIR) spectroscopy can also be applied to research the DACs. The other unmentioned characterization methods for DACs can be seen in other reviews [54,55].

## 4. Electrocatalytic Applications

In this chapter, recent developments for DACs in water-splitting applications are summarized. The relationships between the structure of DACs and electrochemical properties is discussed, which provides a new inspiration for the design of DACs.

### 4.1. Hydrogen Evolution Reaction

Electrochemical hydrogen evolution reaction provides a cost-efficient and sustainable method to generate high-purity H_2_ effectively, which is an important component of developing green energy technologies [56,57,58,59,60]. The widely accepted electron-transfer steps of HER in both acid and alkaline electrolytes are summarized in Table 1.

According to the reaction pathway, the HER in alkaline solutions includes Tafel, Heyrovsky, and Volmer steps, in which Tafel slopes are 30 mV dec^−1^, 40 mV dec^−1^, and 120 mV dec^−1^, respectively [61]. The reaction rate of HER is determined by the ΔG_H*_ (the adsorption-free energy of hydrogen on the catalyst surface). The closer the ΔG_H*_ to zero, the more conducive to achieving rapid HER kinetics by balancing the adsorption and desorption of H* on the surface [62,63,64]. The ΔG_H*_ mainly relies on the intrinsic electronic properties of the electrocatalysts. Thus, the reasonable design of a DAC catalyst is very crucial.

So far, Pt group metal (PGM) has been widely considered as the state-of-the-art electrocatalyst for HER due to the optimal ΔG_H_* value and rapid reaction kinetics [24]. However, their practical applications are inhibited by the high price and natural scarcity. To solve this problem, PGM-based DACs were researched in HER, including Pt, Pd, and Ru dual atom sites. We will introduce some typical DACs to the HER in different electrolytes.

For example, Sun et al. utilized an atomic layer deposition process to prepare high-quality Pt-Ru dimer structures on nitrogen-doped carbon nanotubes. The electrocatalytic performance of the Pt-Ru dimer was investigated in H_2_SO_4_ by cyclic voltammograms. It shows much higher mass activity (more than 50 times) compared with the commercial Pt/C. Both the XAS spectra and theoretical calculations reveal the formation of the Rt-Ru bond. Detailed studies indicate that the high HER activity can be attributed to the introduction of Pt, which can modulate the electronic structure between Ru and H [65].

In another work, Yu et al. developed a Pt_1_Ru_1_/NMHCS-A DAC, which consists of a Pt-Ru dimer and an activated N-doped mesoporous hollow carbon sphere. The Pt_1_Ru_1_/NMHCS-A has a superior HER activity in an acid solution due to the synergistic effect and electron redistribution effect in the Pt_1_Ru_1_ dimer, which can simultaneously accelerate the H_2_ production. The Pt_1_Ru_1_/NMHCS-A exhibits a quite small overpotential of 22 mV to drive a current density of 10 mA cm^−2^. Meanwhile, a small Tafel slope of 38 mV dec^−1^ implies a Volmer–Heyrovsky mechanism in the HER process. The spectroscopic investigations and theoretical calculations reveal that electron redistribution is promoted by Ru atoms, and the Pt can accumulate protons for the HER process, leading to optimal HER activity [66].

The DACs composed of noble metal and transition metal were also proven to be effective in acidic solutions. Wang et al. report dual single-atom Rh-Fe as an excellent electrocatalyst (FR-NCS) for the HER in 0.5 M H_2_SO_4_, the FR-NCS exhibited an ultralow overpotential of 36 mV at 10 mA cm^−2^ with a corresponding Tafel slope of 26 mV dec^−1^, which is superior to 20 wt% Pt/C and other single-atom electrocatalysts. Experiments and first-principles calculations indicate that the interaction of H on the Rh site is stronger than that of Fe sites. The Rh sites enhance the adsorption and evolution of H, and Fe sites facilitate the charge distribution. The excellent activity of FR-NCS for HER can be attributed mainly to the synergistic effect between Rh and Fe sites [67].

**Figure 5:**
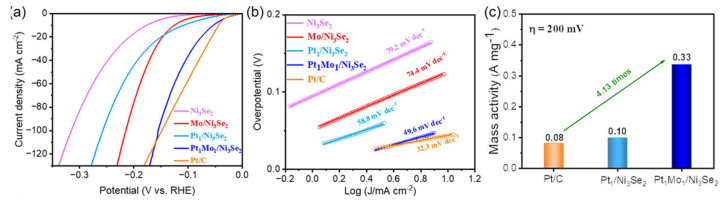
Electrochemical HER performance in 1.0 M KOH. (**a**) Linear sweep voltammetry curves of Ni_3_Se_2_, Mo/Ni_3_Se_2_, Pt_1_/Ni_3_Se_2_, Pt_1_Mo_1_/Ni_3_Se_2_, and Pt/C. (**b**) Tafel slope curves. (**c**) Comparison of mass activity for Pt_1_/Ni_3_Se_2_, Pt_1_Mo_1_/Ni_3_Se_2_, and Pt/C. Reproduced with permission [68]. Copyright 2023 Wiley-VCH GmbH.

The electrocatalytic activities of DACs in alkaline electrolytes have also been researched. For example, Cheng et al. prepared Pt_1_Mo_1_/Ni_3_Se_2_ dual-atom catalysts. As shown in Figure 5a, it only needs 53 mV to reach the current density of 10 mA cm^−2^ in 1 M KOH media, which outperforms the contrast electrocatalysts. The Pt_1_Mo_1_/Ni_3_Se_2_ shows a smaller Tafel slope of 49.6 mV dec^−1^ in Figure 5b, implying the quick reaction kinetics over it. Meanwhile, its mass activity is 4.13 times higher than that of Pt/C at 200 mV (Figure 5c). Combining the experimental results and density functional theory calculations, the introduction of Mo atoms can effectively regulate the hydrogen adsorption energy of Pt sites and boost the HER activity. Generally, the synergistic effect between Pt and Mo sites can realize the charge redistribution, thus optimizing the adsorption of H* and enhancing the electrochemical performance [68]. This work provides a new strategy for the development of DAC, which is compatible with commercial Pt/C in alkaline media.

In another work, Zhang et al. fabricated a bimetallic Ru and Ni co-modified MoS_2_; the as-prepared Ru/Ni-MoS_2_ has excellent HER activity in 1 M KOH solution [69]. It displays an ultrasmall overpotential of 32 mV with the corresponding Tafel slope of 41 mV dec^−1^. Experiments and theoretical calculations unveil that the Ru atoms were anchored on the Ni atoms atop sites, the Ni atoms bonded with S atoms served as a hydrogen acceptor and Ru atoms served as a hydroxyl acceptor, and the synergistic effect accelerated the water dissociation and the HER reaction.

Some non-noble metal-based DACs were confirmed to be excellent HER electrocatalysts in alkaline electrolytes. Rogach et al. prepared dual-atom CoNi on the surface of Ti_3_C_2_T_x_ (CoNi-Ti_3_C_2_T_x_) [70]. The HER performance was evaluated in 1.0 M KOH. It displayed a HER overpotential of 31 mV to deliver 10 mA cm^−2^ and a Tafel slope of 33 mV dec^−1^. The Ti_3_C_2_T_x_ MXene was modified by L-tryptophan molecules so the substrate can stabilize Co and Ni atoms by forming metal-O/metal-N bonds. Efficient electron transfer occurs in CoNi-Ti_3_C_2_T_x_, which lowers the energy barriers of HER.

DACs adapt to various pH values and can benefit the efficient hydrogen production from water. In the study by Fan et al., they developed a dual-atom catalyst consisting of an O-coordinated W-Mo heterodimer embedded in N-doped graphene (NG) [42]. The W_1_Mo_1_-NG DAC enables Pt-like activity and ultrahigh stability for HER in pH-universal electrolytes. The W_1_Mo_1_-NG produced a current density of 10 mA cm^−2^ at an overpotential of 25 mV in 0.5 M H_2_SO_4_ and 67 mV in 1 M KOH. In addition, the Tafel slope of W_1_Mo_1_-NG was 72 mV dec^−1^ and 98 mV dec^−1^ in acid and alkaline electrolytes, respectively. The distinctive W-O-Mo-O-C configuration in W_1_Mo_1_-NG serves as the active site for HER, endowing the electrocatalyst with superior activity and stability.

**Figure 6:**
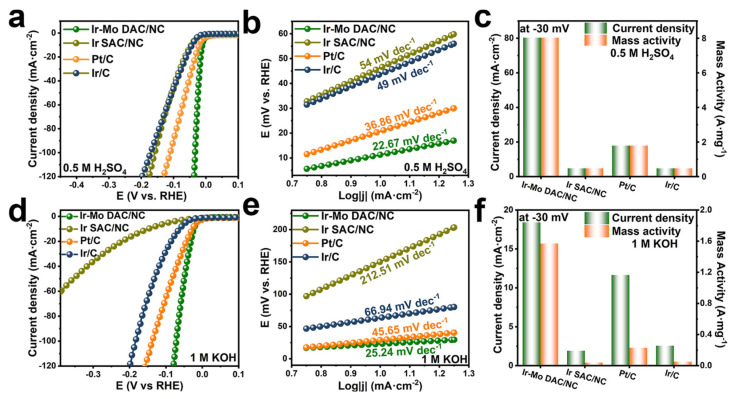
(**a**) LSV curves in 0.5 M H_2_SO_4_, (**b**) Tafel slope for the samples, (**c**) mass activity at 30 mV in 0.5 M H_2_SO_4_ electrolyte for these samples. (**d**) LSV curves in 1 M KOH, (**e**) Tafel slope for the samples, and (**f**) mass activity at 30 mV in 1 M KOH electrolyte. Reprinted with permission from [71]. Copyright 2024 American Chemical Society.

DACs can achieve effective HER across a wide pH span, outperforming the performance of Pt/C. Qu and co-workers prepared N-coordinated Ir-Mo DAC on a carbon matrix [71]. The as-prepared Ir-Mo DAC/NC has an overpotential of 11.3 mV at 10 mA cm^−2^ in 0.5 M H_2_SO_4_, surpassing that of Ir/C and Pt/C (Figure 6a). In this environment, it also achieves a low Tafel slope of 22.67 mV dec^−1^ (Figure 6b). The mass activity of Ir-Mo DAC/NC surpasses the commercial Pt/C and Ir/C catalysts in acidic electrolytes, as displayed in Figure 6c. The HER activity of Ir-Mo DAC/NC was further evaluated in 1 M KOH (Figure 6d–f) to investigate its pH universality. It has an overpotential of 23 mV to deliver a current density of 10 mA cm^2^ with a corresponding Tafel slope of 25.24 mV dec^−1^ and a mass activity of 1569 mA/mg_Ir_, all of which are better than the commercial Ir/C and Pt/C. DFT calculations indicate that the electronic synergy between Ir and Mo modulates the interaction between the intermediates and active sites, arising outstanding electrochemical performance.

In another work by Wang et al., they synthesized NiPt dual atom in situ on porous carbon sponges (NiPt/CMS) as HER self-supporting electrodes [72]. It had an overpotentials of 67 mV in 1 M KOH and 66 mV in 0.5 M H_2_SO_4_ to deliver a current density of 10 mA cm^−2^. The superior HER activity of NiPt/CMS can be attributed to the distinctive structure configuration and strong synergistic effect between Ni and Pt atomic sites.

Table 2 provides a performance summary of the state-of-the-art DACs for HER. Based on the above results, DACs occupy a dominant position in HER, including both acidic electrolytes and alkaline electrolytes.

### 4.2. Oxygen Evolution Reaction

OER process is the half-reaction of electrochemical water splitting relative to HER; it also plays a crucial role in rechargeable metal–air batteries corresponding with ORR.

The OER consists of different four-step electron transfers in both acidic and alkaline electrolytes, as summarized in Table 3. The OER process is more intrinsically sluggish and results in large overpotential when compared with HER [73,74,75]. Meanwhile, the reaction processes are different in various electrolytes with different pH values.

To date, the Fe-, Ni-, and Co-based DACs are the most studied electrocatalysts for OER. An OER in acidic electrolytes is hindered by sluggish kinetics and limited stability. Thus, an OER under alkaline conditions is preferable [76,77,78].

For example, Peng et al. developed atomically dispersed Fe-Ni active sites embedded in a nitrogen-doped carbon substrate (FeNi NPs/NC); the as-obtained FeNi electrocatalyst displays a low overpotential of 270 mV to deliver a current density of 10 mA cm^−2^ for the OER in 1 M KOH. The density functional theory calculation indicates that the rate-determining step to be overcome in bimetallic FeNi SAs/NC is smaller than those of reference samples, suggesting that the synergetic coupling between Fe and Ni atoms can enhance the OER performance. In this work, Fe atoms facilitate the four-electron reaction process, while Ni atoms can modulate the electronic structure of Fe atoms and reduce the energy barrier of the rate-determining step [79].

The interaction between metals and supporting materials can affect the catalytic performance. In the work of Luo et al., they anchored dual metal atoms Ni and Fe via Fe-N_4_ and Ni-N_4_ coordination on nitrogen-doped graphene surfaces (NiFe-DG). The NiFe-DG exhibits high catalytic activity for OER; an overpotential of 358 mV was achieved as 10 mA cm^−2^ with a lower Tafel slope of 76 mv/dec than commercial Pt/C in 1 M KOH [80]. This study uses a supporting material to anchor and stabilize Ni and Fe sites, and the DFT calculations confirm that the introduction of structural defects in the supporting material may tune the active sites’ reactivity.

**Figure 7:**
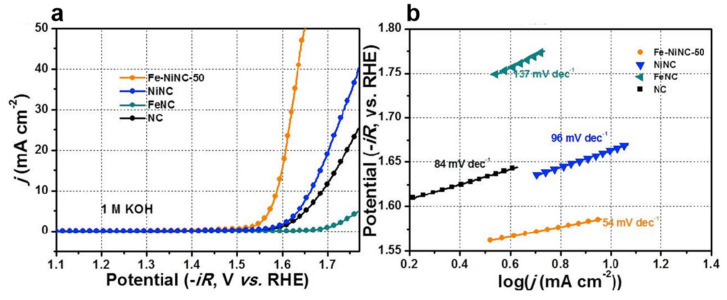
(**a**) LSV for the catalysts in 1 M KOH. (**b**) Tafel plots of the catalysts in 1 M KOH. Reproduced with permission [81]. Copyright 2020 Elsevier B.V.

In another work, Lu et al. fabricated Fe-Ni DACs into nitrogen-doped carbon hollow spheres (Fe-NiNC) as an outstanding electrocatalyst for OER in alkaline electrolytes (Figure 7), which is comparable to the noble metal-based electrocatalysts. A small overpotential of 450 mV can be reached over Fe-NiNC-50 to deliver a current density of 10 mA cm^−2^ in 1 M KOH electrolyte. The Tafel plot for the Fe-NiNC and reference electrocatalysts are displayed in Figure 7b. The Fe-NiNC-50 has the smallest Tafel slope of 54 mV dec^−1^ among all the tested samples. The combined experiments and DFT calculations reveal that the OER mechanism on Fe-NiNC is different from those with single atoms. In the OER process, all the intermediates are inclined to adsorb on the Ni site in the presence of the OH ligand bonding to the Fe. The Fe-Ni pairs have a mutual effect on charge redistribution and enhancing the performance of OER [81].

Some other DACs, including dual-atomic Fe-Ni pairs dispersed in hierarchical porous nitrogen-doped carbon (FeNi-HPNC) [82], Fe and Ni co-anchored on defect-rich porous nitrogen and sulfur carbon frameworks (FeNi-SAs/DNSC) [83], dual-metal single atomic NiFe on N-doped carbon matrix (NiFe-N-C) [84], Fe-Ni dual-atom sites embedded in the N, P co-doped carbon matrix (FeNi-DAC) [85], were also reported as superior OER electrocatalysts in alkaline electrolytes.

The synergetic effect between Fe and Co makes it one of the most promising non-noble metal-based OER electrocatalysts. In the work of Liu et al., they conducted DFT calculations to analyze the activity originating from the synergistic effect between Fe and Co sites with metal–N_4_ configuration. With the guidance of DFT, they developed Fe-Co dual-metal sites (FeCo-NC) through a simple pyrolysis strategy [45]. The OER performance of Fe_3_Co_7_-NC was then evaluated in 0.1 M KOH; it possesses an overpotential of 343 mV at the current density of 10 mA cm^−2^, and the corresponding Tafel slope is 69 mV dec^−1^. In another work, Li et al. prepared a Co-Fe DACs (Co/Fe-SNC800) and its OER activity was then investigated in 1 M KOH [86]. The overpotential at a current density of 10 mA cm^−2^ was as low as 240 mV, lower than that of the commercial IrO_2_. Detailed experimental and theoretical investigations indicate the Co atoms served as active sites to initiate the activation of the reactants. The electronic interaction between Fe and Co can reduce the adsorption of oxygen species on the Fe sites and further facilitate the formation of intermediate species. In other works, various strategies and substrates were applied to construct Fe-Co DACs. For example, Fe-Co polyphthalocyanine compounds were embedded on a hollow, carbonized shell to synthesize FeCo-N_4_/HCS [49]; Fe-Co sites were immobilized into ZIF-8-derived carbon matrix to form Fe_1_Co_3_-NC-1100 [87]; and FeCo dual sites were dispersed into N/P co-doped carbon to generate FeCo-NPC [88]. These electrocatalysts were proven to have superior OER performance in alkaline electrolytes.

CoNi DACs are other candidates for OER in alkaline solutions. For example, Co-Ni DACs were dispersed on N-doped porous carbon frameworks as outstanding OER electrocatalysts (CoDNi-N/C) [89]. The synergetic effect between Co/Ni-N-C bonds originates from high OER activity, which shows an overpotential of 310 mV to generate a current density of 10 mA cm^−2^, surpassing the reported Pt/C electrocatalysts. In the work of Lou et al., the combination of experimental and theoretical calculations unveiled that heteronuclear NiCo can lower the energy barrier for OER [90]; the Ni/Co dual sites on nitrogen-doped carbon (a-NiCo/NC) were synthesized and had a small overpotential of 252 mV in 0.1 M KOH at a current density of 10 mA cm^−2^. Furthermore, the a-NiCo/NC exhibited a low Tafel slope of 49 mV dec^−1^, and the excellent OER activity and kinetics originated from the delicate design of the dual-metal sites. In another work, Li et al. constructed a Co_1_-PNC/Ni_1_-PNC electrocatalyst via the formation of a Co_1_-PN and Ni_1_-PN planar configuration [91]. The as-obtained CoNi DAC displayed a high OER activity in 1 M KOH; the overpotential to deliver the current density of 10 mA cm^−2^ was 390 mV, which is close to that of a commercial RuO_2_ catalyst. The direct coordination of P and N with Co/Ni DAC can modulate the binding interaction of intermediates, thus guaranteeing better OER performance.

Except for Fe, Co, and Ni, some other elements introduced into DACs have also proven effective. For example, Chen et al. regulated the Co d-orbital electron configuration by preparing Ir-Co DAC. The as-developed IrCo-N-C has an exceptional OER activity with a small overpotential of 330 mV at 10 mA cm^−2^ in 0.1 M KOH [92]. In another work, Hao et al. designed nitrogen-doped carbon-supported Co and Ru DAC for outstanding OER performance under alkaline conditions. The Co/Ru DACs display a low potential of 338 mV to reach 10 mA cm^−2^ current density in OER. DFT calculations certificate that Ru can optimize the adsorption energy of Co sites; this work provides a more reasonable strategy for designing DACs [93]. In a study by Li et al., a novel FeMn-DSAC, which comprised Fe-N_4_ and Mn-N_4_, was synthesized [94]. It requires an overpotential of 405 mV to reach a current density of 10 mA cm^−2^ with a corresponding Tafel slope of 96 mV dec^−1^, revealing its favorable OER activity.

Substantial research efforts have also been devoted to developing DACs that have excellent activity and are durable for acidic OER. For example, in the work of Zhao et al., the OER performance of Ru/Co-N-C-800 °C and contrast samples were evaluated in both alkaline and acidic solutions [46]. Notably, Ru/Co-N-C-800 °C with dual-atom sites significantly improve OER activity compared to Ru-N-C-800 °C and commercial RuO_2_, suggesting that the active sites in Ru/Co-N-C-800 °C are Ru-N_4_ sites, which can be further optimized by introducing of Co-N_4_ sites. The overpotential for Ru/Co-N-C-800 °C to reach a current density of 10 mA cm^−2^ are 276 and 232 mV in 1 M KOH and 0.5 M H_2_SO_4_, respectively. The Tafel slope was 67.5 mV dec^−1^ in 0.5 M H_2_SO_4_, implying Ru/Co-N-C-800 °C has superior activity in both alkaline and acidic media. The detailed investigation suggests that RuCo dual sites possess high surface coverage of OH* and improve OER performance.

In another work, Zang et al. synthesized a pyrolysis-free Ni/Fe bimetallic electrocatalysts (CPF-Fe/Ni) as a superior OER electrocatalyst with a simple method and low preparation cost [95]. Its overpotential at 10 mA cm^−2^ is 23 mV and 42 mV in 0.5 M H_2_SO_4_ and 1 M KOH, respectively. The introduction of Ni atoms weakened the adsorption of intermediates, leading to an optimized energy level and enhanced reaction activity. However, the reaction kinetics in the neutral electrolyte (1 M KCl) are slow due to the low concentration of adsorbed reactants.

In summary, OER with a four-electron transfer process has sluggish kinetics and results in a large overpotential. Table 4 summarizes the recently reported DACs for OER. The most widely researched electrocatalysts for OER are the noble metal oxide (e.g., RuO_2_, IrO_2_), but the high costs hinder their practical applications [96]. DACs with intriguing properties are expected to realize outstanding OER performance. However, how to balance the competition between the rate-limiting steps of reversible oxygen reduction and oxygen evolution reaction remains a challenge.

## 5. Summary and Perspective

Facing the energy crisis and environmental problems, the development of catalysts brings new opportunities. DACs have drawn extensive attention due to their maximum atom utilization, atomic active sites, adjustable electronic configuration, and outstanding catalytic performance. In this paper, we summarized the preparation strategies, characterizations, and catalytic applications of DACs in water-splitting reactions reported in recent years.

Even though DACs exhibit efficient activity during water-splitting reactions, they still face challenges in several aspects:

(1) Despite great progress in the synthesis of DACs, the accurate control of the atomic structure and uniform dispersion are still in the initial stage. For example, impurities (e.g., SACs and nanoclusters) are inclined to generate during high-temperature pyrolysis. Meanwhile, the accurate amount of metal precursors is difficult to control; single atoms or metal clusters generate inevitably at the same time. The question of how to synthesize DACs in which one metal atom is merely bonded to another remains unresolved. Thus, it is necessary to combine different synthetic strategies and develop new synthetic methods. Additionally, DACs consist of main group elements worth exploring. The design of heteronuclear DACs, which combines transition and main-group metals, can uncover the synergistic effect between these elements.
(2) Different supports for DACs will bring different electronic structures and enhance performance. MOFs, ZIFs, covalent organic frameworks, and g-C_3_N_4_ are widely applied to serve as supports for DACs; there is plenty of room for optimization. An interesting aspect of metal supports, such as metallene [97,98], which has a two-dimensional nanosheet morphology, may offer cooperative electronic interactions with guest metal atoms. Meanwhile, the stability and catalytic properties of DACs supported by different supports need to be further explored.
(3) Different characterization techniques can identify the structure of DACs, such as HAADF-STEM and XAS. For example, the HAADF-STEM can observe the DACs with an atomic-level resolution, and XAS can analyze the local structure of the DACs regarding the metal–metal interaction, oxidation state, bond length, and coordination environment. At the same time, it is difficult to monitor the structure change and evolution during the reaction in situ constantly. The real active sites under working conditions may be different from those of the ex situ conditions. More advanced in situ/operando equipment should be considered, which can provide more information about the structure–activity relationship and guide the design of DACs.
(4) The stability timescale of electrocatalysts for water-splitting in industrial applications is usually months or even years, which is far beyond the laboratory research lever. Even the Pt/C catalyst with excellent performance can only be used for 40 h [99]. DACs face the risk of the agglomeration and leaching of metal atoms in actual operation (operating under high current densities); the controlled synthesis of high-quality and stable DACs remains a major obstacle.
(5) Due to the shortage of freshwater resources, the electrolysis of seawater has become a research hotspot. Therefore, it is important to develop robust and inexpensive DACs for seawater electrolysis reactions.

Overall, this review systematically summarizes the recent advances of DACs for HER and OER electrocatalysis. DACs are promising candidates for water-splitting reactions due to their unique structures and performance. During the past years, a wide range of SACs, metal oxides, and two-dimensional materials was systematically investigated for the water-splitting reaction. Table 5 and Table 6 provide some of them for HER and OER as comparisons for DACs. There is still a long way to go before DACs are widely applied in practical applications, and continuous efforts should be devoted to this highly exciting research field.

## Figures and Tables

**Table 1:** Electron-transfer steps of HER.

Acid	Alkaline
* + H^+^ + e^−^ → H*	* + H_2_O + e^−^ → OH^−^ + H*
* + H^+^ + e^−^ + H* → H_2_	H_ad_ + H_2_O + e^−^ → OH^−^ + H_2_
2 H* → H_2_	2 H* → H_2_

**Table 2:** Summary of HER performance for reported DACs electrocatalysts.

Electrocatalysts	Overpotential to Reach the Current Density of 10 mA cm^−2^	Tafel Slope	Ref.
NiCo DASs/N-C	189 mV in 1 M KOH 260 mV in 0.5 M H_2_SO_4_	72.5 mV dec^−1^ in 1 M KOH and 82.4 mV dec^−1^ 0.5 M H_2_SO_4_	[29]
FeMo@CoNi-OH/Ni_3_S_2_	89 mV in 1 M KOH and 177 mV in 0.5 M H_2_SO_4_	92.2 mV dec^−1^ in 1 M KOH and 89.3 mV dec^−1^ in 0.5 M H_2_SO_4_	[31]
PtNi-NC	30 mV in 0.5 M H_2_SO_4_	27 mVdec^−1^ in 0.5 M H_2_SO_4_	[32]
Fe/Ni-N-PCS	360 mV in 1 M KOH	57 mV dec^−1^ in 1 M KOH	[34]
CuCo/NSC1	159 mV in 1 M KOH	75.9 mV dec^−1^ in 1 M KOH	[38]
W_1_Mo_1_-NG	24 mV in 0.5 M H_2_SO_4_ and 67 mV in 1 M KOH	30 mV dec^−1^ in 0.5 M H_2_SO_4_ and 45 mV dec^−1^ in 1 M KOH	[42]
Ru/Co-N-C-800 °C	19 mV in 1 M KOH and 17 mV in 0.5 M H_2_SO_4_	27.8 mV dec^−1^ in 1 M KOH and 23.3 mV dec^−1^ in 0.5 M H_2_SO_4_	[46]
Pt_1_Ru_1_/NMHCS-A	22 mV in 0.5 M H_2_SO_4_	38 mV dec^−1^ in 0.5 M H_2_SO_4_	[66]
FR-NCS	22 mV in 0.5 M H_2_SO_4_	26 mV dec^−1^ in 0.5 M H_2_SO_4_	[67]
Pt_1_Mo_1_/Ni_3_Se_2_	53 mV in 1 M KOH	49.6 mV dec^−1^ in 1 M KOH	[68]
Ru/Ni-MoS_2_	32 mV in 1 M KOH	41 mV dec^−1^ in 1 M KOH	[69]
CoNi-Ti_3_C_2_T_x_	31 mV in 1 M KOH	33 mV dec^−1^ in 1 M KOH	[70]
Ir-Mo DAC/NC	11.3 mV in 0.5 M H_2_SO_4_ and 23 mV in 1 M KOH	22.67 mV dec^−1^ in 0.5 M H_2_SO_4_ and 25.24 mV dec^−1^ in 1 M KOH	[71]
NiPt/CMS	66 mV in 0.5 M H_2_SO_4_ and 67 mV in 1 M KOH	72 mV dec^−1^ in 0.5 M H_2_SO_4_ and 98 mV dec^−1^ in 1 M KOH	[72]

**Table 3:** Electron-transfer steps of OER.

Acid	Alkaline
H_2_O → OH* + H^+^ + e^−^	2H_2_O → OH* + 3OH^−^ + e^−^
OH* → O* + H^+^ + e^−^	OH* + OH^−^ → O* + H_2_O + e^−^
O* + H_2_O → OOH* + H^+^ + e^−^	O* + OH^−^ → OOH* + 3 e^−^
OOH* → O_2_ + H^+^ + e^−^	OOH* + OH^−^ → O_2_ + H_2_O + e^−^

**Table 4:** Summary of OER performance for reported DACs electrocatalysts.

Electrocatalysts	Overpotential (E_j10_) to Deliver a Current Density of 10 mA cm^−2^	Tafel Slope	Ref.
FeNi_jns_/NC	440 mV in 1 M KOH	106 mV dec^−1^ in 1 M KOH	[30]
FeMo@CoNi-OH/Ni_3_S_2_	160 mV in 1 M KOH	73.5 mV dec^−1^ in 1 M KOH	[31]
Ni-N_4_/GHSs/Fe-N_4_	390 mV in 0.1 M KOH	81 mV dec^−1^ in 0.1 M KOH	[33]
CuCo/NSC2	339 mV in 1 M KOH	45.3 mV dec^−1^ in 1 M KOH	[38]
NiFe-CNG	270 mV in 0.1 M KOH	60 mV dec^−1^ in 0.1 M KOH	[44]
Fe_3_Co_7_-NC	343 mV in 0.1 M KOH	69 mV dec^−1^ in 0.1 M KOH	[45]
Ru/Co-N-C-800 °C	232 mV in 0.5 M H_2_SO_4_ and 276 mV in 1 M KOH	67.5 mV dec^−1^ in 0.5 M H_2_SO_4_ and 65.7 mV dec^−1^ in 1 M KOH	[46]
Pt_1_Mo_1_/Ni_3_Se_2_	53 mV in 1 M KOH	49.6 mV dec^−1^ in 1 M KOH	[68]
FeNi NPs/NC	270 mV in 1 M KOH	56.84 mV dec^−1^ in 1 M KOH	[79]
NiFe-DG	358 mV in 1 M KOH	67 mV dec^−1^ in 1 M KOH	[80]
Fe-NiNC	450 mV in 1 M KOH	54 mV dec^−1^ in 1 M KOH	[81]
FeNi-HPNC-2	360 mV in 0.1 M KOH	83 mV dec^−1^ in 0.1 M KOH	[82]
FeNi-SAs/DNSC	350 mV in 1 M KOH	55 mV dec^−1^ in 1 M KOH	[83]
NiFe-N-C	323 mV in 0.1 M KOH	36 mV dec^−1^ in 0.1 M KOH	[84]
FeNi-NPC HT	321 mV in 0.1 M KOH	62.9 mV dec^−1^ in 0.1 M KOH	[85]
Co/Fe-SNC800	240 mV in 1 M KOH	42.97 mV dec^−1^ in 1 M KOH	[86]
FeCo-N_4_/HCS	391 mV in 1 M KOH	78.52 mV dec^−1^ in 1 M KOH	[49]
Fe_1_Co_3_-NC-1100	349 mV in 0.1 M KOH	99.93 mV dec^−1^ in 0.1 M KOH	[87]
FeCo-NPC	317 mV in 0.1 M KOH	53.8 mV dec^−1^ in 0.1 M KOH	[88]
CoDNi-N/C	360 mV in 0.1 M KOH	72 mV dec^−1^ in 0.1 M KOH	[89]
a-NiCo/NC	252 mV in 1 M KOH	39 mV dec^−1^ in 1 M KOH	[90]
Co_1_-PNC/Ni_1_-PNC	390 mV in 1 M KOH	117 mV dec^−1^ in 1 M KOH	[91]
IrCo-N-C	330 mV in 0.1 M KOH	79 mV dec^−1^ in 0.1 M KOH	[92]
Co/Ru SAs-N-C	338 mV in 0.1 M KOH	Unknown	[93]
FeMn-DSAC	405 mV in 0.1 M KOH	96 mV dec^−1^ in 0.1 M KOH	[94]
CPF-Fe/Ni	201 mV in 0.5 M H_2_SO_4_ and 194 mV in 1 M KOH	23 mV dec^−1^ in 0.5 M H_2_SO_4_ and 147 mV dec^−1^ in 1 M KOH	[95]

**Table 5:** Summary of HER performance for some reported electrocatalysts.

Electrocatalysts		Overpotential to Reach the Current Density of 10 mA cm^−2^	Tafel Slope	Ref.
SACs	Pt@CoS	28 mV in 1 M KOH	31 mV dec^−1^ in 1 M KOH	[13]
	W-SAC	85 mV in 1 M KOH	53 mV dec^−1^ in 1 M KOH	[14]
	Pt_1_/OLC^h^	38 mV in 0.5 M H_2_SO_4_	36 mV dec^−1^ in 0.5 M H_2_SO_4_	[15]
	Ru ADC^e^	18 mV in 1 M KOH	41 mV dec^−1^ in 1 M KOH	[20]
Metal oxide	RuO_2_/Co_3_O_4_	57 mV in 1 M KOH	48.85 mV dec^−1^ in 1 M KOH	[7]
	CoO/Fe_3_O_4_	220 mV in 1 M KOH	73 mV dec^−1^ in 1 M KOH	[57]
	ZnCo_2_O_4_@CoMoO_4_	114 mV in 1 M KOH	114 mV dec^−1^ in 1 M KOH	[58]
	Mo-NiO/Ni nanopores	34 mV in 1 M KOH	49 mV dec^−1^ in 1 M KOH	[61]
2D materials	NiFe-MOF-74	195 mV in 1 M KOH	136 mV dec^−1^ in 1 M KOH	[59]
	MoS_2_/NiS_2_	62 mV in 1 M KOH	50.1 mV dec^−1^ in 1 M KOH	[60]
	RuMn	20 mV in 1 M KOH and 18 mV in 0.5 M H_2_SO_4_	32.2 mV dec^−1^ in 1 M KOH and 41.2 mV dec^−1^ in 0.5 M H_2_SO_4_	[97]
	PdNi	59 mV in 1 M KOH	108 mV dec^−1^ in 1 M KOH	[98]

**Table 6:** Summary of OER performance for some reported electrocatalysts.

Electrocatalysts		Overpotential to Reach the Current Density of 10 mA cm^−2^	Tafel Slope	Ref.
SACs	Ag_1_/IrO_x_	224 mV in 0.5 M H_2_SO_4_	50 mV dec^−1^ in 0.5 M H_2_SO_4_	[16]
	Ru-N-C	267 mV in 0.5 M H_2_SO_4_	52.6 mV dec^−1^ in 0.5 M H_2_SO_4_	[17]
	Mo-CoOOH	249 mV in 1 M KOH	60.5 mV dec^−1^ 1 M KOH	[18]
	Ir-NiCo_2_O_4_ NSs	240 mV in 0.5 M H_2_SO_4_	60 mV dec^−1^ in 0.5 M H_2_SO_4_	[19]
Metal oxide	NiFeO_x_-P	255 mV in 1 M KOH	27.07 mV dec^−1^ in 1 M KOH	[73]
	Fe-Mo_5_N_6_/MoO_3_-550	201 mV in 1 M KOH	50.5 mV dec^−1^ in 1 M KOH	[74]
	Co_3_O_4_-VCo	262 mV in 1 M KOH	60.5 mV dec^−1^ in 1 M KOH	[75]
	Ni/Co3O4	311 mV in 1 M KOH	43 mV dec^−1^ in 1 M KOH	[76]
2D materials	Ce-MOF@Pt	340 mV in 1 M KOH	47.9 mV dec^−1^ in 1 M KOH	[9]
	NiVRu-LDH	190 mV in 1 M KOH	83 mV dec^−1^ in 1 M KOH	[10]
	CoP/Ti_3_C_2_ MXene	280 mV in 1 M KOH	95.4 mV dec^−1^ in 1 M KOH	[77]
	LSC/K-MoSe_2_	230 mV in 1 M KOH	79 mV dec^−1^ in 1 M KOH	[78]

## Data Availability

No new data were created or analyzed in this study. Data sharing is not applicable to this article.
